# Supplementary material for: Achieving ZT=2.2 with Bi-doped n-type SnSe single crystals
Source: Nat Commun. 2016 Dec 12;7:13713. doi: 10.1038/ncomms13713 (PMC5160008; doi:10.1038/ncomms13713)
Supplement: Supplementary Information — Supplementary Figures 1-8 and Supplementary Note 1 [file ncomms13713-s1.pdf]

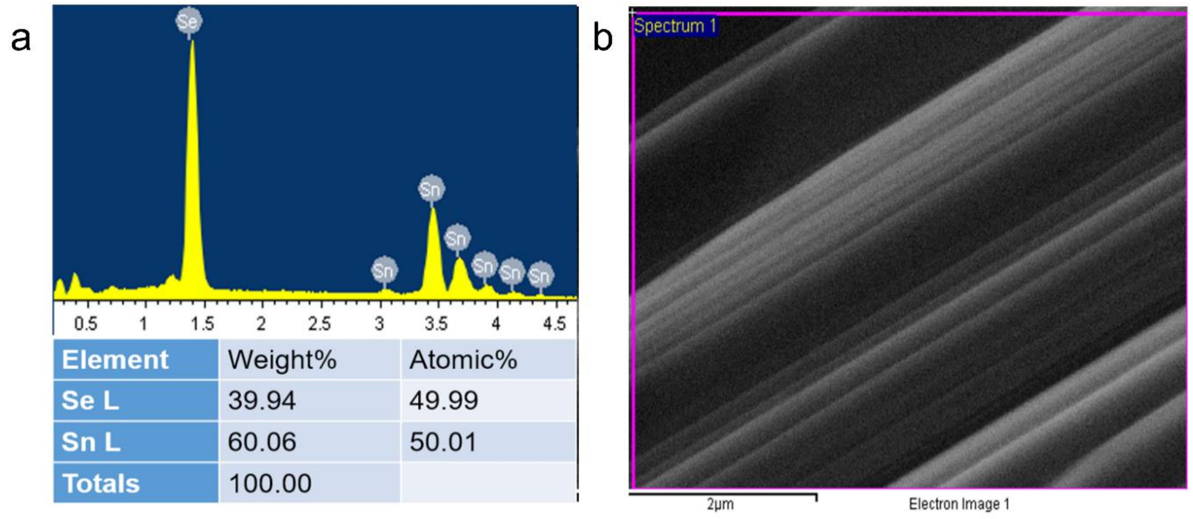

Element	Weight%	Atomic%
Se L	39.94	49.99
Sn L	60.06	50.01
Totals	100.00	

Supplementary Figure 1. Energy dispersive spectrometer (EDS) measurement

(a) (EDS) measurement data indicates the stoichiometry of Sn and Se to be 1:1. (b) FE-SEM image, indicating the layered structure of SnSe.

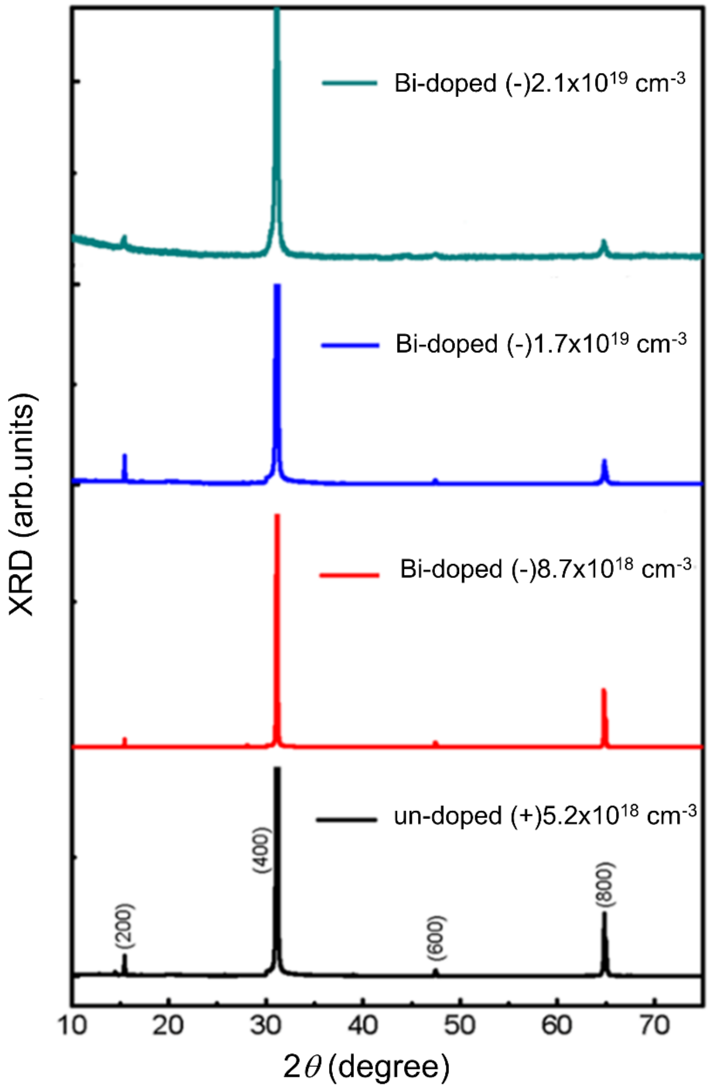

Supplementary Figure 2. X-Ray diffraction patterns

XRD patterns of un-doped and Bi-doped SnSe single crystals, which were cleaved along **a**-axis.

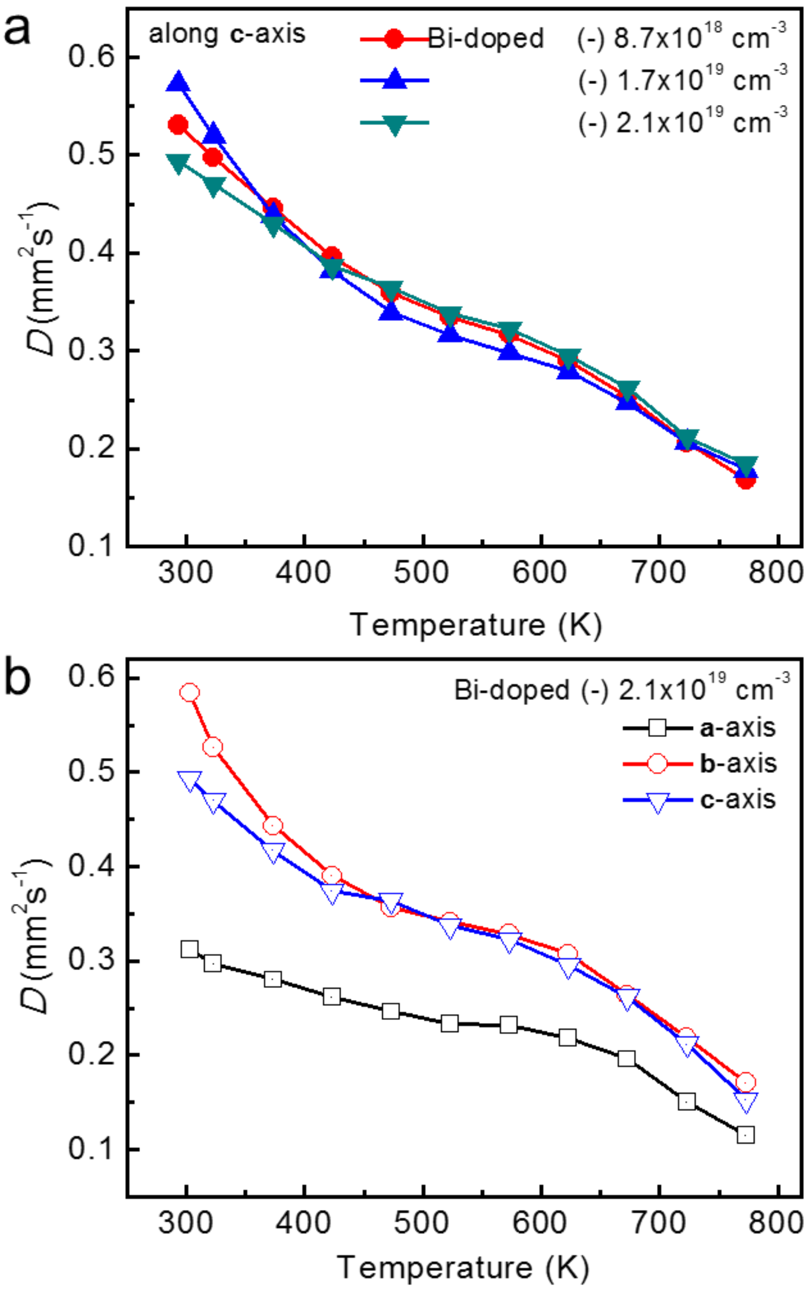

Supplementary Figure 3. Thermal diffusivity

(a) Bi-doped SnSe single crystals. (b) along **a**, **b**, and **c** axes of the sample with a carrier concentration of 2.1×10^{19} cm^{-3} at 773 K.

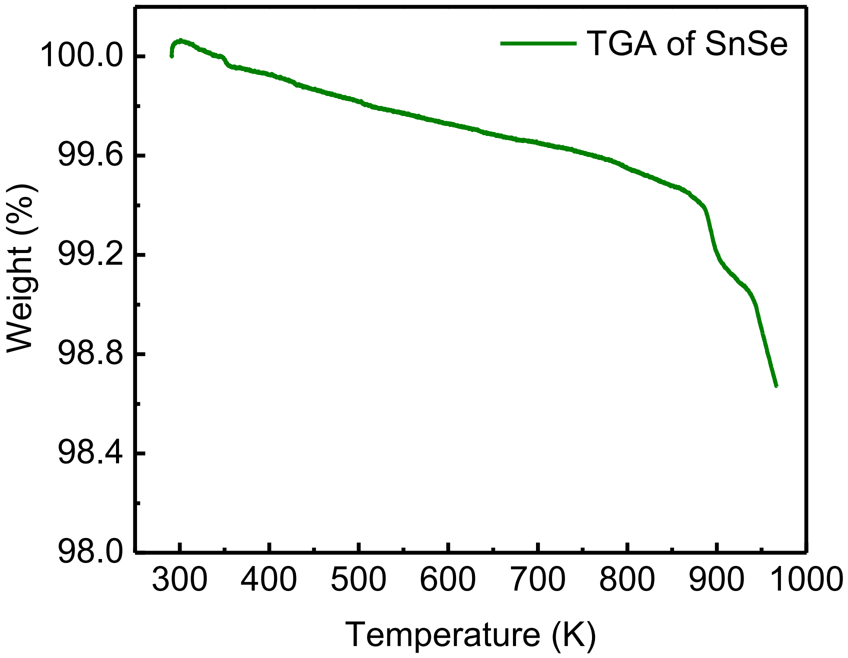

Supplementary Figure 4. Thermogravimetric analysis (TGA) of SnSe single crystal

TGA measurement result shows a drop in weight percent of SnSe at around 873 K.

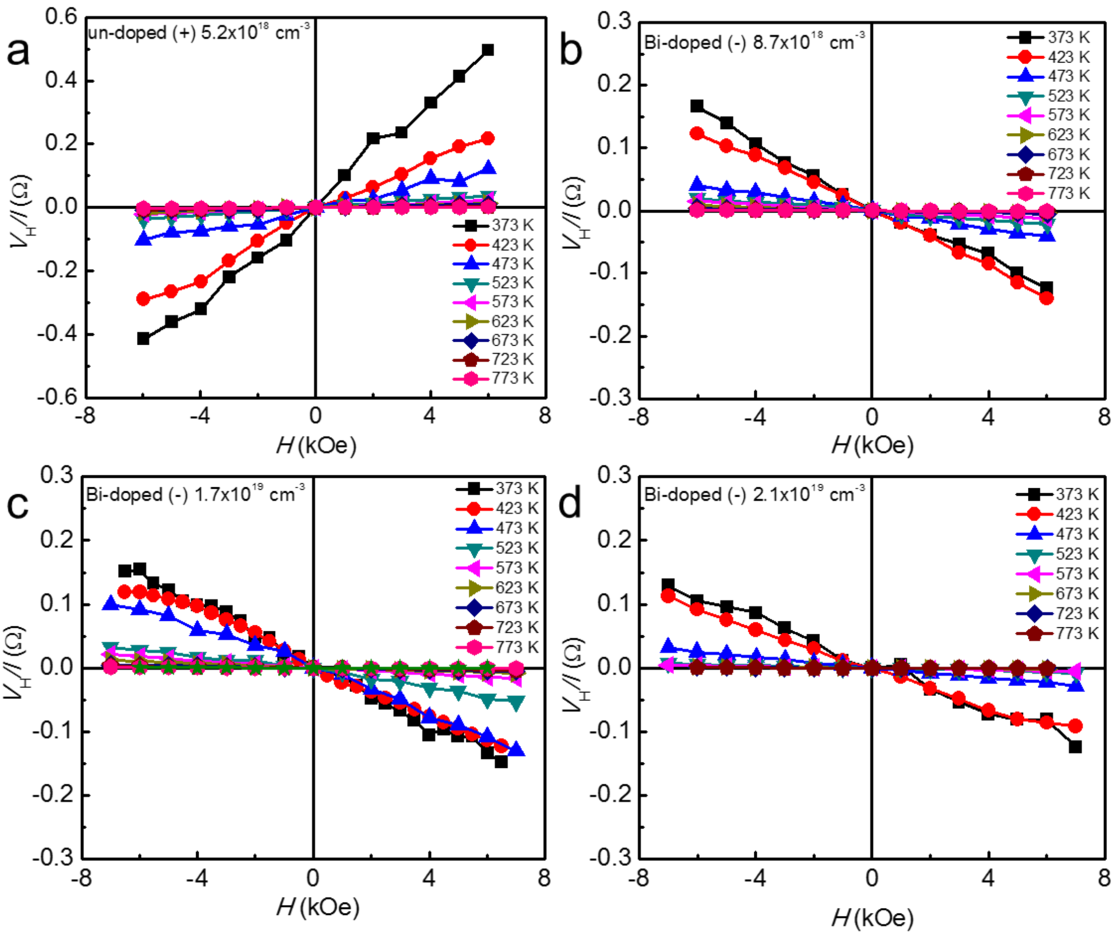

Supplementary Figure 5. Hall resistance vs. magnetic field

(a) un-doped and (b-d) Bi-doped SnSe single crystals with carrier concentration of 8.7×10^{18} cm^{-3}, 1.7×10^{19} cm^{-3}, 2.1×10^{19} cm^{-3}, respectively. We determined the carrier concentrations from the linear fitting of slope. Un-doped sample shows positive slope, indicating dominant hole conduction with the carrier density $+5.2 \times 10^{18}$ cm^{-3} at 773 K.

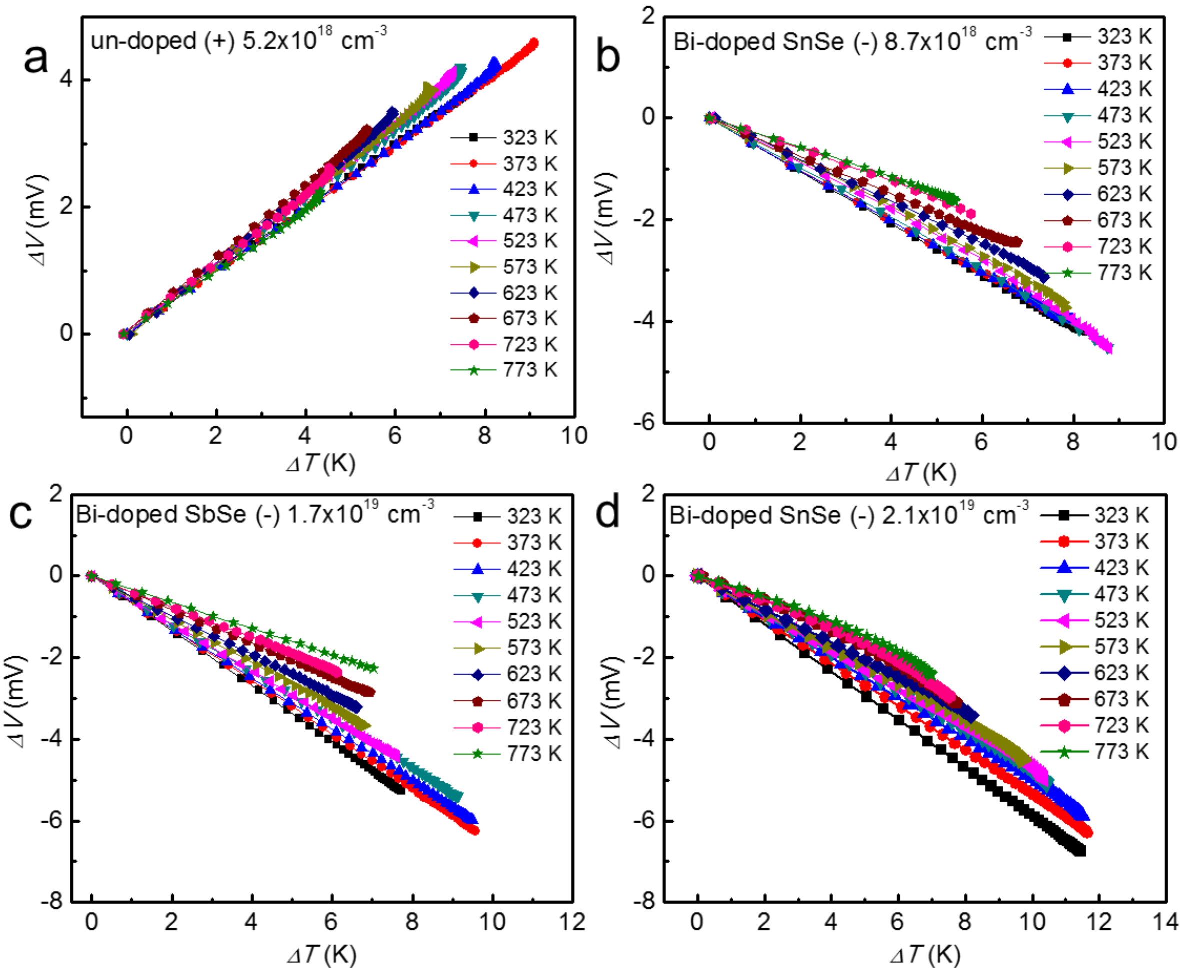

Supplementary Figure 6. Seebeck coefficient determination

Thermoelectric voltage *ΔV* as a function of temperature gradient *ΔT* of un-doped and Bi doped SnSe single crystal.

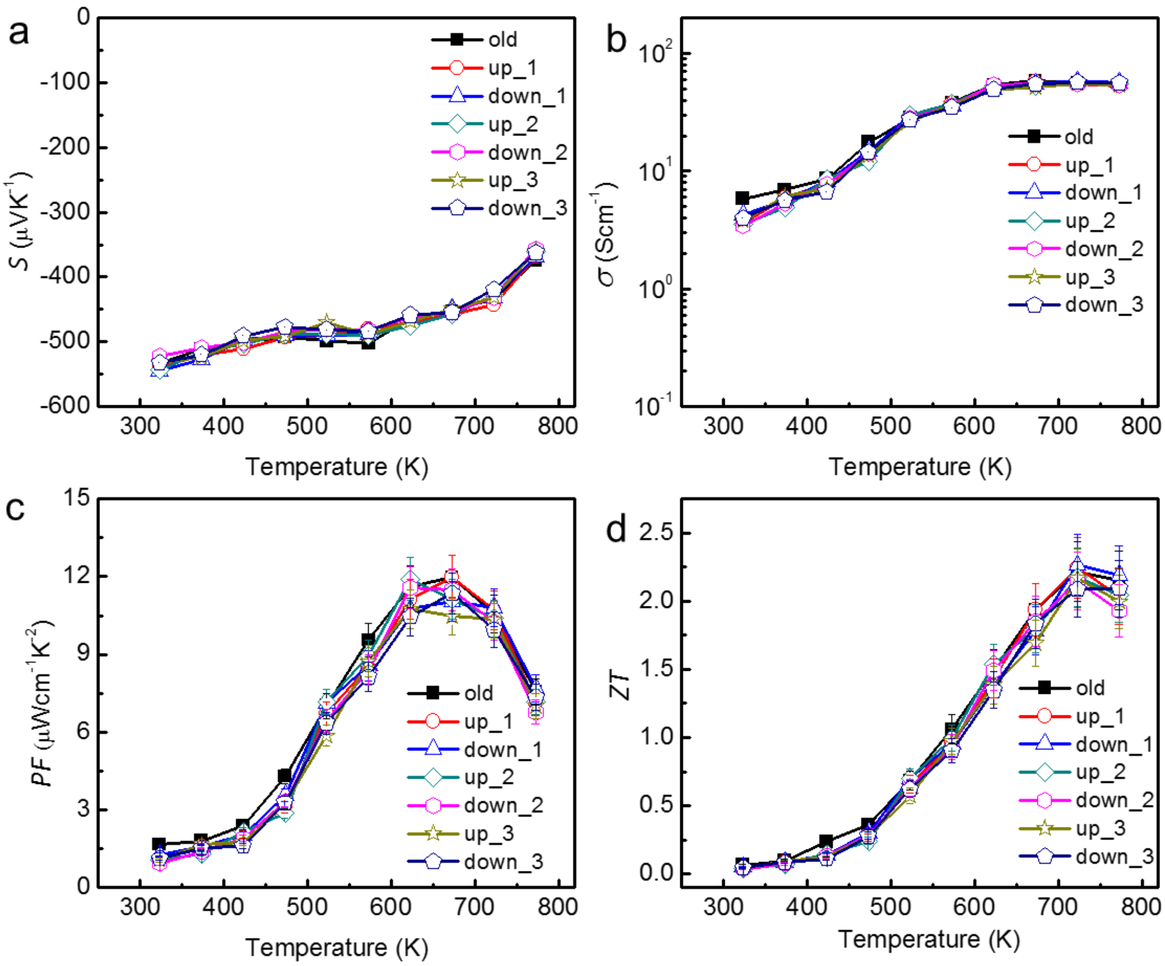

Supplementary Figure 7. Three continuous thermal cycles measurements

Temperature dependent (a) Seebeck coefficients, (b) electrical conductivities, (c) thermoelectric power factors, and (d) *ZT* of Bi-doped SnSe sample with carrier concentration of 2.1×10^{19} cm^{-3} at 773 K along b crystal axis. We performed the transport measurements with three continuous thermal cycles in the temperature range of 323 - 773 K. Old indicates the data in the manuscript for comparison.

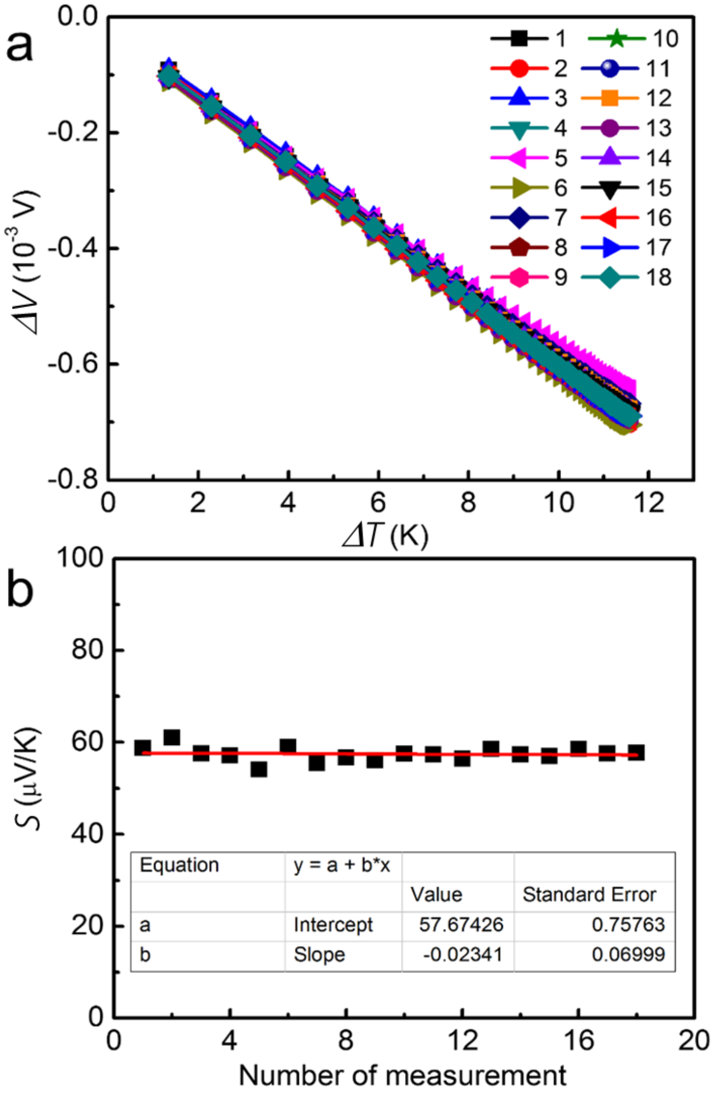

Supplementary Figure 8. Confirm the accuracy of Seebeck measurement system

(a) Thermoelectric voltage ΔV as a function of temperature gradient ΔT; the linear slopes indicate the Seebeck coefficients of polycrystalline Bi. (b) Seebeck coefficient as a function of number of measurement; red line is linearly fitted line using Origin program. Fitting parameters in inset box indicate the average of Seebeck coefficients for 18 times measurements and standard error, 1.3%.

Supplementary Note 1. How to determine measurements error levels.

We determined the error levels of three transport properties (electrical conductivity, Seebeck coefficient, and thermal conductivity) as below.

- For electrical conductivity, $\sigma = \frac{1}{\rho} = \frac{l}{Rab}$ where ρ is resistivity, *R* is resistance, *l* is length of sample, and *a* and *b* are width and height of sample.

Average of electrical conductivity

$$\langle\sigma\rangle = \frac{\langle l\rangle}{\langle a\rangle\langle b\rangle\langle R\rangle} \quad (1)$$

Error of σ is $\frac{\Delta\sigma}{\langle\sigma\rangle} = \left\{\frac{\Delta l}{\langle l\rangle} + \frac{\Delta a}{\langle a\rangle} + \frac{\Delta b}{\langle b\rangle} + \frac{\Delta R}{\langle R\rangle}\right\}$ (2)

Sample sizes; *l* = 2 mm; a = 2 mm; b = 0.3 mm, and *R* = *V/I* (Keitley model 2400 series with error less than 0.1%);

$\Delta l = \Delta a = \Delta b = 0.01\ mm$ then

$$\frac{\Delta\sigma}{\langle\sigma\rangle} = \left\{\frac{0.01}{2} + \frac{0.01}{2} + \frac{0.01}{0.3}\right\} = 0.043 \quad (3)$$

Error for electrical conductivity measurement is 4.3%.

- For Seebeck coefficient measurements. To determine error for Seebeck coefficient, we used a Bi sample whose Seebeck coefficient is well known; i.e. we measured the Seebeck coefficients of polycrystalline Bi for 18 times as shown in Supplelentary Figure 8. We determined Seebeck coefficients from the slope of thermoelectric voltage *ΔV* vs. temperature gradient *ΔT* curve. The measured Seebeck coefficient of polycrystalline Bi is -57.7 ± 0.76 $\mu V.K^{-1}$. Standard error from the linear fitting is 1.3%. Note that the Seebeck coefficient of Bi single crystal is -51.4 $\mu V.K^{-1}$ along perpendicular to the three fold axis and -102.7 $\mu V.K^{-1}$ along parallel to the three fold axis.[3] The Seebeck coefficient of the reported polycrystalline Bi is -60 $\mu V.K^{-1}$ at 300 K (ref. 4). The slight difference between the measured and reported values are sample quality such as garin size, purity, etc.

- For thermal conductivity measurement, we used Laser Flash Apparatus LFA 457 MicroFlash measurement system with the error of ± 3%.
- Power factor (*PF*) is calculared by Seebeck coefficient and electrical conductivity as below:

$$PF = S^2\sigma \tag{4}$$

So *PF* error is estimated:

$$\frac{\Delta PF}{\langle PF\rangle} = \left(\frac{\Delta S}{\langle S\rangle} + \frac{\Delta S}{\langle S\rangle} + \frac{\Delta\sigma}{\langle\sigma\rangle}\right) = (0.013 + 0.013 + 0.043) = 0.069 \tag{5}$$

- ZT error is estimated:

$$\frac{\Delta ZT}{\langle ZT\rangle} = \left(\frac{\Delta S}{S} + \frac{\Delta S}{S} + \frac{\Delta\sigma}{\sigma} + \frac{\Delta\kappa}{\kappa}\right) \tag{6}$$

$$= (0.013 + 0.013 + 0.043 + 0.03) = 0.099$$

Power factor and *ZT* error in our measurements are 6.9% and 9.9%, respectively. Thus, we added error bars in the figures
